# Reslip of a Previously Fixed Slipped Upper Femoral Epiphysis with an Associated Vitamin D Deficiency

**DOI:** 10.1155/2014/528257

**Published:** 2014-02-17

**Authors:** Aziz Ul Haque, Benjamin Bloch, Alwyn Abraham

**Affiliations:** University Hospitals of Leicester, Infirmary Square, Leicester LE1 5WW, UK

## Abstract

Slipped upper femoral epiphysis (SUFE) is a relatively common adolescent hip disorder that represents a biomechanical instability of the proximal femoral growth plate. A link between vitamin D deficiency and SUFE has emerged in recent years; however, we present a unique case of a 10-year-old girl who presented with a reslip of a previously fixed SUFE with an associated vitamin D deficiency.

## 1. Introduction

Slipped upper femoral epiphysis (SUFE) is a common adolescent hip disorder that represents a biomechanical instability of the proximal femoral growth plate. This usually leads to a posteroinferior slip of the epiphysis. It usually presents with hip pain, progressing to a limp and sometimes a leg length discrepancy. If not detected and treated it can lead to avascular necrosis, degenerative hip disease, and gait abnormalities. The overall incidence of SUFE in the United States is 10.8 per 100,000 children and is three times more common in boys than girls [1, 2].

The exact pathology of this condition is still unknown; however, the typical presentation is of an obese adolescent. SUFE has been associated with endocrine abnormalities such as hypothyroidism, hypogonadism, and growth hormone deficiency [3, 4]. Vitamin D deficiency is a common nutritional deficiency in children and has been associated with bone growth as well as bone mineral density of adolescent children [5]. There has only been one case report in the US that has suggested a link between vitamin D deficiency and SUFE [6].

This paper presents a case of a 10-year-old girl who presented with a reslip of a previously fixed SUFE and a vitamin D deficiency.

## 2. Case Report

In 2010, a 10-year-old girl presented with left thigh pain. Radiographs revealed evidence of significant slippage of the left upper femoral epiphysis and a possible early slip on the right. She had no significant past medical history and was not obese.

The patient underwent bilateral single screw fixation. The result was satisfactory with no operative complications. She made good progress following this with a pain-free period for almost 2 years. She was followed up twice yearly and radiographs were unremarkable.

In 2012 she came to clinic complaining of increasing pain in the right groin. She was walking with a Trendelenburg gait and on examination she had an irritable hip with fixed external rotation and impingement sign positive on the right side. The initial slip in the left side had fused fully on repeat radiographs. However, on the right side the upper femoral epiphysis had reslipped despite being fixed with a cannulated screw. Her Southwick angle had increased from 10 degrees in August 2011 to 25 degrees in May 2012. She was advised to mobilise nonweight bearing through her right leg with crutches. She was investigated for endocrine and nutritional abnormalities which highlighted a severe vitamin D deficiency. This was treated with high dose supplementation. During her 18 months of followup she has responded well to treatment. The pain has settled and there have been no further slips. However due to a residual deformity a femoral valgus osteotomy is planned.

## 3. Discussion

The reslip of a previously fixed and stable upper femoral epiphysis with a coexisting vitamin D deficiency and no other endocrine abnormalities makes our case unique. There is a case report [6] that attempts to link vitamin D and SUFE; however, their patient had a complicated postoperative course, a high BMI, and other nutritional deficiencies. In addition they reported that their patient did not achieve a satisfactory outcome from the initial surgery.

Although the exact role of vitamin D in SUFE is unknown, there are studies that suggest that an association does exist. In 2004, Brown [7] reviewed the national database for SUFE in the United States and found a possible link between it and vitamin D deficiency. This was based on an observation of seasonal variation in incidence along with a variation in latitude and skin pigmentation. A study by Jingushi et al. [8] measured vitamin D levels of 13 patients presenting with SUFE and found that 7 of them were severely deficient.

Vitamin D has a key role to play in bone formation, particularly at growth plates during adolescence. It is responsible for matrix mineralisation, which predominantly occurs in the proliferative and hypertrophic zones of the growth plate [9, 10]. Histological studies show that abnormality and subsequent slippage in SUFE occur in the same zones suggesting that vitamin D is involved in its pathogenesis [11, 12].

Additional studies are required to explore the association of vitamin D and SUFE. Currently, we know that vitamin D plays an important role in bone development and small studies have shown that children with SUFE tend to be deficient in vitamin D. It may therefore be beneficial to measure vitamin D in the initial workup of patients presenting with SUFE. This may help in the early detection of a vitamin D deficiency and optimisation of our treatment of SUFE.
